# Is extremely low frequency pulsed electromagnetic fields applicable to gliomas? A literature review of the underlying mechanisms and application of extremely low frequency pulsed electromagnetic fields

**DOI:** 10.1002/cam4.5112

**Published:** 2022-08-05

**Authors:** Mengqian Huang, Parker Li, Feng Chen, Zehao Cai, Shoubo Yang, Xiaohong Zheng, Wenbin Li

**Affiliations:** ^1^ Cancer Center, Beijing Tiantan Hospital Capital Medical University Beijing China; ^2^ Clinical Medicine Shanghai Jiao Tong University School of Medicine Shanghai China

**Keywords:** electromagnetic therapy, extremely low frequency pulsed electromagnetic fields, gliomas, tumor‐specific frequencies

## Abstract

Gliomas refer to a group of complicated human brain tumors with a low 5‐year survival rate and limited therapeutic options. Extremely low‐frequency pulsed electromagnetic field (ELF‐PEMF) is a specific magnetic field featuring almost no side effects. However, the application of ELF‐PEMF in the treatment of gliomas is rare. This review summarizes five significant underlying mechanisms including calcium ions, autophagy, apoptosis, angiogenesis, and reactive oxygen species, and applications of ELF‐PEMF in glioma treatment from a clinical practice perspective. In addition, the prospects of ELF‐PEMF in combination with conventional therapy for the treatment of gliomas are reviewed. This review benefits any specialists, especially oncologists, interested in this new therapy because it can help treat patients with gliomas properly.

## Graphical abstract

Extremely low‐frequency pulsed electromagnetic field (ELF‐PEMF), a specific magnetic field, is regarded as a potential adjuvant therapy for gliomas. This review summarizes significant underlying mechanisms and prospects of EMF‐PEMF on gliomas.

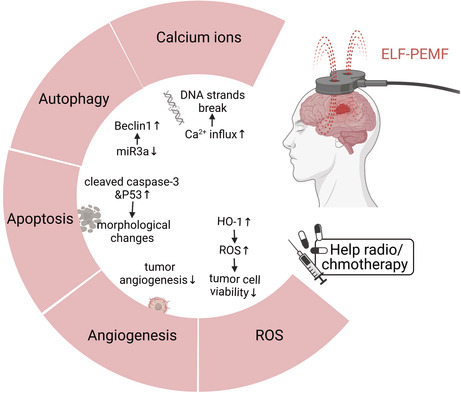

## 1 INTRODUCTION

Gliomas are primary brain malignancies that are derived from glial or precursor cells, accounting for approximately 80.8% of malignant brain tumors, and approximately 25.1% of all central nervous system tumors.^1^ Among the deadliest forms of brain cancers in adults, glioblastoma (GBM) is the most aggressive diffuse glioma with a short median survival of 14.4 months after standard therapy.^1^, ^2^, ^3^ The therapeutic options available to patients with gliomas include surgery, radiotherapy (RT), and chemotherapy; however, these treatments are not as effective as expected considering the anatomical position and self‐renewing tumor stem cells of gliomas.^4^, ^5^, ^6^ For instance, chemotherapy is insufficient after penetrating the blood–brain‐barrier (BBB). Moreover, self‐renewing tumor stem cells of gliomas lead to a poor prognosis after surgery. Thus, therapeutic options for those patients are limited currently.

Over the past decades, researchers have explored new therapies for glioma, such as tumor‐treating fields (TTFields)^7^; the application of magnetic field (MF) and electric field (EF) has become the focus of oncology. EF is widely used in TTFields and plays a therapeutic role in GBM by influencing tumor cell mitosis.^8^, ^9^ However, the mechanisms and therapeutic function of MF remain ambiguous.

Magnetic field and EF are closely related according to the law of electromagnetic induction. Faraday's law states that the time rate of change of an MF generates a potential difference (or electric potential) in the space wherever the MF changes, which means MF of time‐varying characters could generate an EF and induce internal currents in brain tissues. That is to say, MF and EF might have a similar mechanism such as generating internal currents.^10^

Charged particles exist among DNA, proteins and cells, for example, DNA contains phosphate residues that are negatively charged, and K^+^ is the dominant positive ion in cells. Hence, applying an MF externally could affect internal particles, and may thereby alter biological processes in tumor cells. In view of the high penetrability of MF in high‐resistance structures like the skull and its association with EF,^10^, ^11^ some researchers are keen to know the therapeutic potential of MF in gliomas.

Electromagnetic field (EMF) is an MF generated by electric currents, it has been also regarded as a justifiable part of physiotherapy in tumor treatment, of which their clinical use has been of wide concern for the provision of a noninvasive, safe, and complementary method for glioma treatment^11^ In contrast to electrical stimuli that may lead to mild‐to‐moderate dermatitis due to dermal exposure to electrode patches or allergic etiology,^8^ EMF could avoid such side effects by using coils that are not attached to body.^12^

Electromagnetic field is subdivided into pulsed EMF (PEMF) and continuous EMF, with the former being more advantageous than the latter because compared with continuous EMF, PEMF produces signals that could be perceived by brain more easily and delivers a large amount of energy in short bursts at a lower level of average energy.^13^, ^14^ Existing studies have revealed the potential of PEMF in treating depression,^15^, ^16^ osteoarthritis,^17^ rheumatoid arthritis,^18^ repairing tendons,^19^ and preventing ulcer formation in diabetes patients.^20^ Moreover, PEMF has been used to treat breast cancer^21^ and melanoma.^22^

However, PEMF has limited clinical use because its optimal parameters, such as frequencies, intensities, exposure times even waveforms, remain uncertain. For example, the frequency of PEMF is associated with its tissue penetrability and consequent biological effects, and PEMF has been found to be effective when its frequency ranges from 0.16 to 480 Hz and the intensity ranges from 0.6 to 250 mT.^23^ In this review, we focus on extremely low‐frequency PEMF(ELF‐PEMF), a subdivision of PEMF with frequencies between 0 and 300 Hz,^24^ which has the potential to penetrate the skull^10^, ^25^ and inhibit the growth of glioma cell lines.^26^, ^27^

Electromagnetic field at high frequencies, such as radio frequencies EMF (frequencies at 3 kHz–300 MHz),^28^, ^29^ generates thermal damage, whereas other types of EMF including ELF‐PEMF are generally believed to cause negligible thermal damage,^30^, ^31^, ^32^ suggesting that the mechanisms through which ELF‐PEMF affects tumor cells should be further investigated.

In this review, we summarize the studies regarding the mechanisms of ELF‐PEMF and review the potential synergic therapeutic effects of ELF‐PEMF on glioma, hoping to obtain an improved understanding of its underlying mechanisms and provide new insights into glioma treatment.

## 2 CALCIUM IONS

The relationship between Ca^2+^ and gliomas began to draw attention^33^ when T‐type channels were found to be expressed in the proliferative stage of the cell cycle^34^ and that the overexpression of T‐type channels could induce the proliferation of glioma cells.^35^ Blocking T‐type channels Cav3.2, a target in gliomas, could reduce the survival rate of GBM cells and their resistance to temozolomide (TMZ).^36^ Plus, calcium‐activated potassium channels are found to be overexpressed in gliomas and are directly related to tumor growth and invasiveness.^37^ However, ELF‐PEMF appears to act differently because it activates the T‐type calcium ion pathway, and has an impact on the membrane. During this process, Ca^2+^ ions are observed to move from the outside to the inside of cells via Ca^2+^ channels. The increase in calcium concentration under exposure to different ELF‐PEMFs stems the growth of tumor cells,^22^, ^27^, ^38^ which may involve different downstream signaling pathways.

Buckner^22^ exposed various cancer cell lines or normal cells to ELF‐PEMF (25–6 Hz, 2–10 μT) at 1 h/day for 5 days as listed in Table 1. The proliferation of tumor cells in the treatment group was suppressed by 30%–50% compared with that of the tumor cells in the control group. Interestingly, non‐malignant cell lines were unaffected. The difference was attributed to the inappropriate intracellular levels of calcium resulting from the aberrant expression of T‐type Ca^2+^ channels in many malignant cell lines.^39^ They proved that a specific ELF‐PEMF affected T‐type channels related to Ca^2+^ influx. In addition, the ELF‐PEMF pattern^22^ reached similar results in mice injected with melanoma cells.

**TABLE 1:** Summary of main findings of reviewed studies

Target	Cell types	ELF‐PEMF	Exposure time and other characteristics	ELF‐PEMF effects	Conclusions	References
Calcium ions	B16‐BL6, HSG, MDA‐MB‐231, MCF‐7, HeLa, HBL‐100, and HEK293 cells	(25–6 Hz, 2–10 μT) Epileptic seizure wave pattern	In vitro:1 h/day for 1, 3, or 5 days In vivo: 3 h/day until the mice were sacrificed and tumors dissected	Ca^2+^ influx↑, Cancer cell proliferation↓	ELF‐PEMF could inhibit tumor cell proliferation and tumor growth in mice, which might result from activation of T‐type calcium channel	(22)
	MCF7 cells and MCF10 cells	(20–50 Hz, 2–5 mT)	30, 60, or 90 min/day for 1, 2, or 3 days	ELF‐PEMF (20 Hz, 3 mT, 1 h/day) is most cytotoxic to MCF7 cells related to DNA strands break	DNA strands break occurred downstream of calcium‐stimulated caspase activation; the ELF‐PEMF which is cytotoxic to cancer cells is not damaging to normal cells	(38)
Autophagy	SH‐SY5Y, SK‐N‐SH, U87‐MG, and T98G cell lines	(75 Hz, 2 mT)	1,3,6,12 or 24 h	miR‐30a↓, BECN↑, especially after 1 h exposure	ELF‐PEMF could affect autophagy by regulating miR‐3a, and the pattern that induces autophagy might protect neurons simultaneously	(48)
Apoptosis	U87 cell lines	(100 Hz, 10 mT), (10 Hz, 5 mT), (50 Hz, 10 mT), (50 Hz, 5 mT) Square wave	2, 4, 24 h	Cell viability↓, P53↓, caspase‐3↓ after PEMF(10 Hz, 5 mT); cell viability↑ after PEMF (100 Hz, 10 mT)	Specific ELF‐PEMF might induce cells to apoptosis via affecting caspase‐3 and P53, and frequencies and amplitudes have a bearing on its effects	(26)
Angiogenesis	C3H/HeJ mice implanted with murine 16/C mammary adenocarcinoma cells	(60 Hz with 0, 10, 15, or 20 mT) (120 Hz, 10–20 mT) Semi‐sine wave	3–80 min either once or twice a day for 12 days	1. ELF‐PEMF gave the maximum anti‐angiogenic effect at (15 mT, 10 min/day); maximum suppression of tumor growth at 20 mT for 10 min twice a day. 2. The extent of vascularization↓, tumor necrosis↑ after exposure	ELF‐PEMF could reduce the vascular (CD31+) volume fraction and increase the necrotic volume of the tumor, but its effects on tumor regression remain poorly evidenced	(23, 68)
Epigenetic modulation	T98G	(75 Hz, 2 mT)	1 h every 2 days for 8 days Group.1 PEMF (1 h), 24 h without PEMF, TMZ (10 μM, 24 h); Group. 2 TMZ (10 μM, 24 h) immediately after PEMF(1 h)	Apoptotic indices↑ in group.1; expression of miR‐17‐3p, miR‐21‐5p and miR‐421‐5p↓ in group.2	A combination of TMZ and ELF‐PEMF could retard tumor proliferation epigenetically	(85)
ROS	T47D	(100 or 217 Hz, 0.1 mT)	24, 48 or 72 h	Ros↑ at 217 Hz for 72 h; Actin level↑ at ELF‐PEMF (100, 217 Hz, 0.1mT)	ELF‐PEMF led to oxidative cellular stress and morphological changes in actins	(21)
	U87 and T98G cells	(100 Hz, 10 mT) Square or sinusoidal waves	72, 96, 120, or 144 h Combined with TMZ(100 μM)	Expression of P53, Bax, and Caspase‐3, HO‐1↑, ROS↑; expression of Bcl‐2 and Cyclin‐D1↓	ELF‐PEMF may enhance the apoptotic effects of TMZ through redox regulation	(63)
	UTSCC15, A549, DLD1 and MiaPaca2 cell lines	(30 Hz, max.35 μT) Half‐wave‐shaped sinusoidal	8 min, 1 or 24 h Combined with cetuximab, cisplatin or gemcitabine treatment, and/or RT	Cell survival↓, DNA strand breaks↑ after ELF‐PEMF combined with RT; the radiosensitizing effect was abolished with a 24 h interval between RT and ELF‐PEMF	The radiosensitization produced by ELF‐PEMF depends on its duration, intensity, and interval between radiation and ELF‐PEMF treatment. And it is associated with increased ROS and subsequent DNA strand breaks	(78)
	SH‐SY5Y cells	(75 Hz, 2 mT)	18, 36, 54, or 72 h	HSP70↑, MnSOD↑, ROS↓	ELF‐PEMF led to increasing MnSOD and decreasing ROS, which showed its cytoprotective effect related to dose and time	(79, 80)
			4 times/week, 10, 15, 30 min each	ELF‐PEMF prevented overexpression of HSP70; MnSOD↑, ROS↓		
	U87 cell line	(100 Hz, 10 mT) Square wave	120 or 144 h Combined with TMZ(100 μM)	SOD activity↑, the concentration of Malondialdehyde and Ca^2+^↑ under co‐treatment.	ELF‐PEMF catalyzed apoptosis and differentiation induced by TMZ.	(27)
ARs	PC12 and U87MG	(75 Hz, 1.5 or 3 mT)	24 h Combined with Cl‐IB‐MECA (100 nM)	A_2A_ARs, A_3_ARs↑, NF‐kB↓, and caspase‐3↑ in tumor cells	ELF‐PEMF augmented the anti‐tumor effects of A_3_ARs	(64)
	Rat cerebral cortex and cortical neurons		2, 4 or 6 h	The density of A_2A_ARs↑ after ELF‐PEMF	ELF‐PEMF could regulate expression and function of ARs in neurons and have the potential to protect neurons by affecting A_2A_ARs	(97)

Abbreviations: ↑ indicates increased or activated; ↓indicates decreased or inactivated; ARs, adenosine receptors; Cl‐IB‐MECA, 2‐chloro‐N6‐(3‐iodobenzyl) adenosine‐5′‐N‐methyl‐uronamide; ELF‐PEMF, extremely low frequency pulsed electromagnetic field; h, hour; HO‐1, heme oxygenase‐1 gene; HSP, heat shock proteins; min, minute; MnSOD, Mn‐dependent superoxide dismutase; ROS, reactive oxygen species; RT, radiotherapy; TMZ, temozolomide.

Crocetti^38^ corroborated a hypothesis that DNA breaks in tumor cells are related to the elevation of intracellular Ca^2+^. Malignant cell lines and human non‐tumorigenic cells (Table 1) were exposed to ELF‐PEMF (20–50 Hz, 2–5 mT), for 30–90 min/day for up to 3 days. Malignant cells were vulnerable to ELF‐PEMF (20 Hz, 3 mT), and this pattern offered mild protection to non‐tumorigenic cells simultaneously. DNA break‐ups prior to cell death and downstream of calcium‐stimulated caspase activation were discovered, and the changes detected in mitochondrial metabolism were found to be related to changes in calcium concentration.

Overall, ELF‐PEMF showed the potential to affect Ca^2+^ in tumors,^22^, ^27^, ^38^ as Ca^2+^ pathway has been proven to be related to glioma growth and invasiveness.^34^, ^35^, ^37^ The role of Ca^2+^ ions in ELF‐PEMF might be another topic worth researching in studies on gliomas.

## 3 AUTOPHAGY

Autophagy, a degradative process, occurs in most cells at low basal levels, it maintains homeostasis, and its regulation is closely related to tumorigenesis pathways.^40^, ^41^ Its role in cancer treatment remains controversial.^42^ Meanwhile, studies on its functional relevance in the formation and progression of gliomas have focused mainly on GBM, a type of glioma and the most aggressive primary brain tumor.

Autophagy might be involved in both promotion and inhibition of GBM progression. It has been observed to participate in the mediation of cell death in GBM by oncolytic adenovirus^43^ and rapamycin,^44^ and its activation potentially impairs the migration and invasion of GBM.^45^ However, available researches also indicate that autophagy might impair the efficacy of chemotherapy,^46^ and inhibiting autophagy stems the development of and induces senescence of GBM.^47^

Marchesi^48^ illustrated that autophagy in human neuroblastoma cell lines SH‐SY5Y, SK‐N‐SH and GBM‐derived cell lines U87‐MG and T98G affected gene expression that might be induced by ELF‐PEMF (75 Hz, 2 mT) via the modulation of specific regulatory miRNA sequences. A previous study^48^ indicated that ELF‐PEMF with a specific pattern could decrease miR‐3a levels in GBM cells. Moreover, miR‐3a could target BECN1, a Beclin1 coding gene, to repress Beclin1 expression.^49^ Beclin1 is a positive regulator of autophagy and functions as a tumor suppressor in GBM,^50^, ^51^ which might be associated with the poor prognosis of patients with GBM.^52^ This means that specific ELF‐PEMF can cause tumor death by activating autophagy.

Interestingly, protective effects were observed in human neuroblastoma cells,^48^ which are known as neuronal‐like cells.^53^ The ELF‐PEMF pattern that induced the autophagy of tumor cells might protect neurons simultaneously.

It is of great value to learn the complex role of autophagy in TTFields which may help us study the effects of ELF‐PEMF on gliomas in terms of autophagy. Blocking autophagy attenuated the tumor cell death induced by TTFields,^54^ while another study reported that the upregulation of autophagy in a certain degree response to TTFields could increase the resistance of tumor cells.^55^ More experiments are needed to learn how ELF‐PEMF applications affect autophagy in gliomas.

## 4 APOPTOSIS

ELF‐PEMF is predicted to intrigue apoptosis in cancer cells.^56^, ^57^ Cyclin‐D1, P53, and caspase‐3 are thought to play a pivotal role in pathways concerning apoptosis.^58^, ^59^, ^60^, ^61^, ^62^ This role may account for the effects of ELF‐PEMFs on gliomas.

Akbarnejad^26^ attempted to explore how ELF‐PEMF influences GBM, a malignant and aggressive brain tumor. The author exposed the human GBM U87 cell line to 4 ELF‐PEMF patterns with different frequencies and intensities. The overexpression of cleaved caspase‐3 and P53 proteins after exposure to ELF‐PEMF (100 Hz, 10 mT) or ELF‐PEMF (10 Hz, 5 mT) demonstrated that a specific ELF‐PEMF pattern could promote differentiation and induce apoptosis in U87 cells by affecting the cell cycle or cell division.

Akbarnejad further studied the relationship between PEMF (100 Hz, 10 mT) and TMZ^63^ and proved that PEMF induced the overexpression of caspase‐3 directly and P53 indirectly, with both effects correlated with apoptosis induction.^58^, ^59^, ^60^, ^61^, ^62^ Furthermore, they observed apoptosis‐related morphological changes. In this context, ELF‐PEMF enhanced the anti‐tumor effects of adenosine receptors (ARs) that might be related to P53 and caspase‐3.^64^ These findings indicated that ELF‐PEMF could lead to tumor suppression by influencing apoptosis.

## 5 ANGIOGENESIS

In 1971, Folkman reported that angiogenesis is related to tumor growth, and described the prospects of anti‐angiogenic cancer treatment for the first time.^65^ Drugs for angiogenesis like bevacizumab for glioma treatment have undergone clinical trials. Although overall survival (OS) of patients with GBM was not prolonged in the trials,^66^ epidemiological data implied that OS was prolonged because of bevacizumab's effects,^67^ indicating that anti‐angiogenic treatment has the potential to treat tumors including GBM. ELF‐PEMF is also thought to have vascular effects.^23^

A study^68^ assessed the effects of ELF‐PEMF(60 Hz, 10 min/day with 0, 10, 15, or 20 mT) on C3H/HeJ mice implanted with murine 16/C mammary adenocarcinoma cells, and found that ELF‐PEMF could lead to a reduction in the extent of angiogenesis along with tumor necrosis. Another work^23^ used a device providing ELF‐PEMF(120 Hz, 10–20 mT) to mice implanted with the same cells and determined the vascularization, necrosis, and viable area of tumors. ELF‐PEMF was proposed to be capable of suppressing tumor angiogenesis, which is widely known as an important factor in tumor development.^69^ That ELF‐PEMF could lead to tumor regression remains poorly documented, but this experiment verified its potential to increase the doubling time of tumor growth. Therefore, ELF‐PEMF could retard tumor growth.

## 6 REACTIVE OXYGEN SPECIES

Reactive oxygen species (ROS), including oxygen anions; superoxide; hydroxyl radicals; and peroxides such as hydrogen peroxide (H_2_O_2_), have been regarded to be crucial in cancers, including gliomas.^70^ ROS are thought to contribute to the occurrence and development of cancer by inflicting DNA damage.^71^ Given that cancer cells tend to be highly sensitive to elevated ROS,^72^ the accumulation of ROS to a certain extent can be cytotoxic to cancer cells without affecting normal cells, thus enabling the use of ROS in selective anti‐cancer therapy.^73^, ^74^

Akbarnejad^63^ carried out an experiment to explore the effect of ELF‐PEMF (100 Hz, 10 mT) exposure with 100 μM TMZ on U87 and T98G cells. In the experiment, the heme oxygenase‐1 gene (HO‐1), which generates oxidative cellular stress via ROS production, was found to be overexpressed,^75^ and cell viability decreased as ROS production increased.

A study^21^ observed that actin affected by ELF‐PEMF led to morphological changes in T47D human breast cancer cells while apoptosis was not observed. These effects might be explained by the parameters of ELF‐PEMF including frequency and duration. The study showed that the effects of ELF‐PEMF on cellular growth and ROS generation depended on time and frequency.

A system with ELF‐PEMF(max. 35 μT) was employed in multiple sclerosis with fatigue^76^ and was found to improve organ blood flow.^77^ In another research,^78^ the system was applied to cells from different solid tumors (Table 1). The results illustrated that ELF‐PEMF exerted some effects on glycolysis and TCA cycle pathways and increased ROS levels. The researchers performed a single ELF‐PEMF treatment followed by RT at short intervals and observed the potential of ELF‐PEMF to mediate radiosensitization by increasing the levels of ROS and the subsequent generation of DNA damage to explore the therapeutic implications of these changes.

Two experiments^79^, ^80^ studied the effects of ELF‐PEMF (75 Hz, 2 mT) exposure on the stress and oxidative pathways of human neuroblastoma SH‐SY5Y cells, neuronal‐like cells,^53^ which are often used to determine cellular responses on redox basis.^81^ They observed that ELF‐PEMF could exert a cytoprotective effect by altering redox status, such as by increasing the free radical scavenger superoxide dismutase‐1 enzyme (SOD‐1) and decreasing mitochondrial activity. Furthermore, a growing body of evidence shows that increasing SOD may act as a tumor suppressor.^82^ A further study indicated that ELF‐PEMF treatment could increase the activity of Mn‐dependent superoxide dismutase (MnSOD) which is an essential antioxidant enzyme that is believed to reduce ROS levels.^83^ They summarized that exposure to ELF‐PEMF could act as a catalyst for the major antioxidant enzymatic defense.

All in all, ELF‐PEMF is likely to act on the redox status of cells. Some experiments showed the possibility of its protective effect on normal neurons. Despite different modes, ELF‐PEMF has promising prospects in terms of clinical use.

## 7 OTHERS

### 7.1 Bio‐energy transport

Pang^84^ made an attempt to discover the mechanism of energy transport in protein molecules under EMF. After analyzing Davydov's theory on energy transport, they changed the Hamiltonian and the wave function of systems simultaneously and built a Pang's soliton model on the basis of Davydov's model. They confirmed that Pang's soliton could transport hundreds of amino acid residues and that it varied with the external EMF. That is, EMF could target amino acid residues in protein molecules and influence soliton energy transport, thus affecting bio‐energy. In the article, the term “bio‐energy transport” indicates bio‐energy flows along protein molecules, a process that sustains life activities. Physical models^84^ have been employed to explain this biological process. Such an approach may be a new trend of studies on ELF‐PEMF mechanisms. In addition, the variation in the biological effects of EMFs with strength and direction points out a feasible direction for follow‐up research.

### 7.2 Epigenetic modulation

ELF‐PEMF could mediate the level of miR‐30a to affect autophagy by targeting specific genes.^48^ In 2016, Pasi^85^ used the same ELF‐PEMF (75 Hz, 2 mT) on the chemo‐ and radioresistant human GBM cell line T98G. Their results showed that ELF‐PEMF could decrease miR‐421, miR‐21, and miR‐17 levels, which were found to be overexpressed in tumor cells and to lead to apoptosis resistance in an epigenetic manner.^86^, ^87^, ^88^, ^89^ They also showed that a combination of TMZ and ELF‐PEMF could decelerate tumor proliferation epigenetically.^85^

### 7.3 Adenosine receptors

Recent studies were conducted to examine the influence of ARs changed by ELF‐PEMF. ARs, which are receptors in the G‐protein signaling pathway, are considered to have an effect on cell death and proliferation, and they are classified into A_1_, A_2A_, A_2B_, and A_3_ ARs. In gliomas, A_1_ARs are suggested to impair tumor cell growth and play an anti‐tumor role^90^, ^91^ and may be associated with apoptosis via caspase‐3.^91^ A_2A_ARs have been found in many tumor cells including GBM cells.^92^ Through the underlying influence of ARs on gliomas is poorly understood, the activation of A_2A_ARs may offer considerable protection to neurons.^93^, ^94^ A_2B_ARs are found to contribute to cancer cell proliferation,^95^ while the changes in ARs under ELF‐PEMF require further study. A_3_ARs are considered to be associated with the cell cycle and are highly expressed in tumor cells.^96^ The activation levels of A_3_ARs have been thought to be related to their effects on apoptosis.

In 2011, Varani^97^ performed saturation binding experiments and mRNA expression analysis to identify the influence of ELF‐PEMF (75 Hz, 1.5 mT) exposure on A_2A_ARs in the rat brain and cortex membranes. The density of A_2A_ARs in cerebral cortex membranes was upregulated after 2 h of exposure to ELF‐PEMF, suggesting that ELF‐PEMF might have the potential to protect neurons by affecting A_2A_ARs.

In another study,^64^ human GBM cell lines (U87MG) were exposed to ELF‐PEMF (75 Hz, 1.5 mT) with rat cortical neurons as a comparison. The findings showed that ELF‐PEMF exposure could enhance the expression and density of A_3_ARs. The study^64^ also reported that ELF‐PEMF worked in sync with 2‐chloro‐N6‐(3‐iodobenzyl) adenosine‐5′‐N‐methyl‐uronamide (Cl‐IB‐MECA), an A_3_ARs agonist that could release the inhibition of tumor growth by the NF‐KB pathway to lead to tumor cell apoptosis,^98^ and finally induced G1 cell cycle arrest in tumor cells.^64^, ^99^

In conclusion, ELF‐PEMF influenced ARs and augmented their anti‐tumor effects.

## 8 COMBINATION OF PEMF AND RADIO/CHEMOTHERAPY

The application of ELF‐PEMF in gliomas is drawing attention after the application of TTFields.

A review in 2013^100^ intriguingly suggested that adjuvant EMF treatment may increase RT effectiveness, implying that different cell lines and/or species respond variably to EMF and/or ELF‐MF, but did not specify ELF‐PEMF.^100^ Thus, whether ELF‐PEMF in combination with RT could be applied to cancer treatment, especially glioma treatment, requires further evaluation.

The potential benefits of adjuvant EMF treatment during RT in several cell lines and models, including hepatoma‐implanted mice^101^ and the human lung carcinoma cell line A549,^78^ have also been identified. One study^78^ explored the radiation‐related mechanisms under ELF‐PEMF exposure and proposed that ELF‐PEMF could mediate radiosensitization, which is associated with cancer cell resistance to anticancer drugs, by affecting ROS.^73^

An exploratory study^102^ in 2019 attempted to combine RT and ELF‐PEMF, and exposed epithelial breast cancer cell lines to ELF‐PEMF (50 Hz, 10 mT) then to ionizing radiation. The evaluation of cell cycle progression and free radical production revealed that co‐treatment with ELF‐PEMF before RT was likely to enhance the effectiveness of breast cancer therapy. The combination therapy of gliomas needs further study which could shed light on the new perspectives for glioma treatment.

Given that drug delivery could be promoted by an external trigger such as MF,^103^ ELF‐PEMF application is likely to enhance chemotherapy. A specific ELF‐PEMF pattern has been proposed to be capable of enhancing breast cancer cell therapy by normalizing tissue microcirculation effectively.^21^ This mechanism might also work in gliomas.

After the effects of ELF‐PEMF on U87 cells were identified,^26^ an experiment employed the same device to explore the effect of ELF‐PEMF (100 Hz, 10 mT) exposure with 100 μM TMZ on U87 and T98G cells.^63^ As mentioned in the context, the expression of P53, Bax, and Caspase‐3 increased, whereas that of Bcl‐2 and Cyclin‐D1 decreased, and both of these effects promoted the apoptosis of U87 and T98G cells together. Apoptosis‐related morphological changes were also observed. ELF‐PEMF (100 Hz, 10 mT) exposure was found to strengthen the effects of TMZ in inducing U87 cells to die and differentiate,^27^ thus enabling the combination of ELF‐PEMF with TMZ in GBM treatment. Therefore, ELF‐PEMF could enhance TMZ‐induced apoptosis even when the cell line is TMZ‐resistant, indicating that a combination of ELF‐PEMF and low TMZ doses could achieve the same anticancer efficacy as high TMZ doses while reducing side effects of chemotherapy. The efficacy of the co‐treatment was also corroborated in the experiment, as tumor cell viability decreased evidently after the exposure.

Zhang^104^ and Ding^105^ explored BBB permeability or brain microvascular permeability changes induced by ELF‐PEMF. Despite its mild vascular injury, changes in vascular permeability may allow chemotherapy drugs to cross BBB and act on the brain. If combined with chemotherapy and extracorporeal RT, ELF‐PEMF may help reduce drug doses and improve efficacy. This effect provides new insights into and lays the groundwork for experiments on intracranial tumor treatment.

## 9 DISCUSSION

As considerable achievements have been recorded for various physical therapies, MF therapy, a potential adjuvant therapy, has become widely known, because of its defining features, such as painlessness, invasiveness, and the potential for repeated application. Given that MF can kill cancer cells selectively by influencing cell cycle stages,^38^ its prospects for the treatment of intracranial tumors like gliomas are promising. Several researchers have explored the mechanisms of ELF‐PEMF, in which glioma cell lines are influenced through calcium ions, autophagy, and apoptosis, and suggested that ELF‐PEMF is likely to augment the effects of chemotherapy and RT.

There are several directions worthy of future work:

First, further studies on the appropriate ELF‐PEMF parameters, such as intensities, frequencies, wave forms, and pulse duration, could be conducted to improve the efficiency of ELF‐PEMF.

Second, the type of equipment used for ELF‐PEMF for intracranial tumors is forthcoming. PEMFs are delivered mainly via two means: capacitive coupling and inductive coupling. The former requires direct contact with skin, while the latter does not.^12^ ELF‐PEMF has been used to treat depression by placing a helmet on the head of patients.^15^ It has also been used to treat osteoarthritis by placing sets of coils near the knee^17^ or air‐coil devices that are designed to be non‐contact.^106^ ELF‐PEMF could also be applied by stimulating acupuncture points to reduce peritumoral edema.^107^ Although trials on gliomas are insufficient, the design of appropriate equipment is in the pipeline. Perhaps similar approaches to depression treatment could be taken.

Finally, in vivo experiments, prospective studies, and well‐organized randomized controlled trials remain inadequate. Moreover, the safety of the use of ELF‐PEMF in the long term requires in‐depth investigation.

Due to the development of electromagnetics, the upgrading of devices, and the advancement of the interdisciplinary combination of medicine and engineering science, ELF‐PEMF would be applied successfully in glioma treatment.

## AUTHOR CONTRIBUTION

M.H. searched the literature, drew tables, and drafted the manuscript. P.L. and Z.C. drafted and proofread the manuscript. F.C. guided the writing of the paper. X.Z. and S.Y. assisted in writing the paper. W.L. was responsible for selecting the topic and critically revising important intellectual content. All authors contributed to the article and approved the submitted version.

## CONFLICT OF INTEREST

The authors have no conflicts of interest to report.

## ETHICS STATEMENT

This is a review article based on published literature. Ethical statement is not applicable.

## Data Availability

All data come from published literature or journal articles that have been cited in the manuscript.
